# Measurement of serum short-chain fatty acid concentrations in cattle after oral administration of difructose anhydride III

**DOI:** 10.14202/vetworld.2023.1505-1511

**Published:** 2023-07-24

**Authors:** Topas Wicaksono Priyo, Seiichi Uno, Emiko Kokushi, Katsuki Toda, Hiroshi Hasunuma, Daisaku Matsumoto, Osamu Yamato, Masayuki Ohtani, Oky Setyo Widodo, Dhidhi Pambudi, Masayasu Taniguchi, Mitsuhiro Takagi

**Affiliations:** 1 Department of Clinical Veterinary Science, Joint Graduate School of Veterinary Sciences, Yamaguchi University, Yamaguchi 753-8515, Japan; 2 Department of Reproduction and Obstetric, Faculty of Veterinary Medicine, Universitas Gadjah Mada, Indonesia; 3 Education and Research Center for Marine Resource and Environment Faculty of Fisheries, Kagoshima University, Kagoshima 890-0056, Japan; 4 Shepherd Central Livestock Clinic, Kagoshima 899-1611, Japan; 5 Department of Veterinary Clinical Science, Joint Faculty of Veterinary Medicine, Kagoshima University, Kagoshima 890-0062, Japan; 6 Nippon Beet Sugar Manufacturing Co., Ltd., Obihiro 080-0835, Japan; 7 Division of Animal Husbandry, Faculty of Veterinary Medicine, Universitas Airlangga, Indonesia; 8 Department of Mathematics Education, Faculty of Teacher Training and Education, Sebelas Maret University, Indonesia; 9 Department of Veterinary Clinical Science , Joint Faculty of Veterinary Medicine, Yamaguchi University, Yamaguchi 753-8515, Japan

**Keywords:** cattle, difructose anhydride III, short-chain fatty acid

## Abstract

**Background and Aim:**

We previously reported the mitigation effects of difructose anhydride III (DFA III) on mycotoxins, such as zearalenon and sterigmatocystin, based on the urinary concentrations of these molecules in calves. This study was aimed at evaluating the effects of dietary supplementation of DFA III and the fermented status of DFA III in the intestine by comparing serum levels of short-chain fatty acid (SCFAs) in DFA III-supplemented cattle with those in non-supplemented control cattle.

**Materials and Methods:**

Serum SCFA concentrations were measured in 30 Japanese Black heifers, aged 9–10 months, from two herds, using gas chromatography on days 0 (before DFA III supplementation), 9, and 14 after DFA III supplementation.

**Results:**

A notably different trend was observed for isobutyric acid and enanthic acid, which may reflect the different fermentation status of supplementary DFA III in the intestine. Our results indicate the possibility that this trend observed in the intestinal tract following DFA III administration is associated with changes in the environment of intestinal bacterial flora, which may partially reflect the effects of DFA III supplementation on cattle.

**Conclusion:**

Difructose anhydride III supplementation for at least 2 weeks affects the trend of blood SCFA concentrations in cattle. Our results provide evidence supporting the effects of DFA III on the intestinal environment and intestinal barrier function.

## Introduction

As the use of antibiotics in animal production may contribute to the resistance of human pathogens, alternatives, such as probiotics and prebiotics, have recently been proposed to overcome the challenges associated with the reduction of antibiotic usage [1–3]. Therefore, there is growing interest in the health-promoting effects of prebiotics, such as mannanoligosaccharides [1, 4], fructooligosaccharides [5], lactulose [6], and fermentation products [7]. Prebiotics have been reported to positively influence the bacterial flora of the gastrointestinal tract (by increasing the population of *Bifidobacteria* and *Lactobacillus*), thereby, reducing the incidence of diseases in animals [6, 7]. A recent consensus regarding indigestible saccharides is that they stimulate short-chain fatty acids (SCFAs), including acetic, propionic, and butyric acids, which may improve the intestinal microbiota composition [8, 9].

In addition to the problem of resistant pathogens due to antibiotic abuse, contamination of agricultural commodities with mycotoxins, which are secondary fungal metabolites, is also a major global concern in agriculture and livestock production [10]. Consumption of mycotoxin-contaminated products causes acute and chronic effects in humans and animals; thus, contamination of food, feed, and ingredients by mycotoxins poses considerable health risks [10, 11]. Therefore, mitigation strategies against mycotoxins are required at livestock production sites worldwide. To date, mycotoxin adsorbents (MAs) applied in animal feed generally consist of a mixture containing a mineral clay carrier, yeast cell wall preparations, and, in some cases, enzymes or living microorganisms (probiotics). Recently, direct interactions of the oligosaccharides with intestinal epithelial cells have been reported, which indicates that these oligosaccharides improve and protect the integrity of the intestinal barrier and modulate the immune responses of epithelial cells [12–14]. Difructose anhydride III (DFA III) is a naturally occurring, non-digestible disaccharide in commercially roasted chicory manufactured from inulin through microbial fermentation [15, 16]. We previously reported the efficacy of DFA III supplementation as a prebiotic for the improvement of the health and intestinal microbiota in calves [17, 18]. We showed that it protected the integrity of the intestinal barrier in cattle and can be used as an alternative to protect against chronic low-dose mycotoxin contamination from cattle diets [19, 20]. A previous report with experimental animals (rats) indicated that cecal SCFA concentrations in the DFA III-supplemented group were higher than those in the non-supplemented group under the same feeding conditions [21, 22]. Although a large volume of SCFAs is produced through microbial fermentation of plant cellulose in the rumen, the dynamics of SCFAs in intestinal fermentation following the addition of DFA III as a supplement to cattle feeds are unknown. Because DFAIII is not degraded in the rumen and is fermented by intestinal microorganisms, the increase in SCFA concentrations in the intestinal tract due to the addition of DFA III and the accompanying change in SCFA concentrations in the blood due to absorption from the intestinal tract were hypothesized.

As a first step to clarify the mechanism underlying the effect of DFA III supplementation on cattle, this study was conducted to compare serum SCFA levels in DFA III-supplemented cattle and non-supplemented control cattle and to evaluate the fermented status of DFA III in the intestine.

## Materials and Methods

### Ethical approval

The study was approved by the Institutional Animal Experiment Ethic Committee of Yamaguchi University, Japan (Approval no. 40, 1995 dated 27-03-2017).

### Study period and location

The research was conducted in December 2017 on two Japanese Black cattle farms in Kagoshima Prefecture, Japan.

### Chemical and solvents

Difructose anhydride III was donated by Nippon Beet Sugar Manufacturing Co. Ltd., Obihiro, Japan. We prepared a mixture of short fatty acids-acetic acid, propionic acid, isobutyric acid, butyric acid, 2-methylbutanoic acid, isovaleric acid, valeric acid, caproic acid, 2-methylhexanelic acid, and enanthic acid (Sigma Aldrich, Tokyo, Japan ) to measure SCFAs in the serum samples using gas chromatography-mass spectrometry (GC/MS) according to previous reports on the concentrations of SCFAs in serum samples or rumen fluids of cattle [9, 23, 24].

### Japanese black (JB) cattle herds and sample collection

The details regarding animals and collected serum samples used in the present study were as described in previous studies by Toda *et al*. [19], Sasazaki *et al*. [20]. Briefly, JB female heifers from two beef herds (Herd 1: n = 10; Herd 2: n = 20; approximately 10 months old and weighing 250–300 kg) raised in Kagoshima Prefecture, Japan, were included in this experiment. Herds 1 and 2 consisted of 370 and 500 JB cattle, respectively. Herds 1 and 2 were fed concentrate and rice straw. The composition of the dietary feed provided to the two herds is summarized in Table-1 [19]. Two groups of heifers were randomly selected from each experimental herd and assigned to two treatment groups that differed in feed supplementation as follows: Difructose anhydride III group (Herd 1: n = 5; Herd 2: n = 10) fed 40 g DFA III/days (20 g, each feeding time) mixed with concentrate, and the control group (Herd 1: n = 5; Herd 2: n = 10) fed without DFA III supplementation. The dose of DFA III used in the study is the recommended dose for prevention of hypocalcemia in dairy cows [25, 26], which may affect the functions of the tight junction.

**Table-1:** Composition of feed provided to the two herds was kept for fattening purposes.

Herd	Forage feed, kg	Formula feed						

		Total, kg	Bran, %	Cereal, %	Oil seed meal, %	Other, %	TDN, %	CP, %
Herd 1	Straw 2.0, Timothy grass 2.0	3	24	46	16	14	>70.0	>16
Herd 2	Straw 2.0, Oats 2.0	2	27	56	7	10	>71.5	>14

TDN: Total digestible nutrients, CP: Crude protein. This table is adopted from Toda *et al*. [19].

Blood samples were collected from the jugular vein in silicone-coated tubes after two hours of morning feeding. In previous studies by Toda *et al*. [19], Sasazaki *et al*. [20], although we performed blood sampling at the start of DFA III supplementation (day 0), on days 9 and 14 (day 14, i.e., on the last day of DFA III supplementation) after treatment initiation and on the last day of the experimental period on day 23, the blood samples collected on days 0, 9, and 14 were used for the measurement of SCFA concentrations in the present study during the DFA III-supplemented periods.

All samples were immediately placed in a cooler containing dry ice for protection from light and were transported to the laboratory. Blood samples were centrifuged at 2000× *g* for 10 min at 20–25°C. Serum samples were frozen at −80°C until SCFA concentrations were analyzed.

### Gas chromatography-mass spectrometry analysis of SCFAs in serum samples

The analytical procedures used for measuring the concentrations of SCFAs in the plasma were as described by Furuhashi *et al*. [27]. One milliliter of plasma with fibrin removed was placed into a 2 mL polypropylene (PP) tube with a zirconia ball. Plasma was added to 1 mL of 10% isobutanol and shaken using a shaker (TissueLyser II, Qiagen, Tokyo, Japan). The sample was centrifuged at 21,000× *g* for 5 min, and 675 μL of the supernatant was transferred to another PP tube. 3-methyl pentanoate was used as an internal standard, and 125 μL of 20 mM NaOH and 400 μL of chloroform were added to the solution. The solution was subsequently mixed using a vortex mixer. Four hundred microliters of the upper layer (aqueous phase) was transferred into another PP tube, followed by centrifugation (21,000× *g*, 2 min). A solution of isobutanol (80 μL), pyridine (100 μL), and ultrapure water (70 μL) was added. A boiling stone was placed in the tube to avoid bumping. For derivatization using isobutyl esterification, 50 μL of isobutyl chloroformate was added to the solution in the PP tube. Thereafter, 150 μL of hexane was added to the tube and the mixture was shaken using a vortex mixer. After centrifugation at 21,000× *g* for 2 min, the upper layer was collected and placed in a glass vial. The derived SCFAs were measured using GC/MS (GC-2030 equipped with QP2020 NX and AOC-20i Plus, Shimadzu Corp., Kyoto, Japan). Samples were injected into a DB-1HT column (30 m length, 0.25 mm id, 0.10 μm film thickness, Agilent Technologies, Tokyo, Japan). The injector and detector temperatures were 260°C and 280°C, respectively. The column temperature was increased incrementally as follows: 40°C for 5 min, 180°C at a rate of 10°C/min, and then 310°C at 30°C/min. The temperature was maintained for 3 min.

### Statistical analysis

We examined the differences in time-series trends in blood SCFA concentrations between the control and DFA III-supplemented (DFA III) groups. A linear mixed model analysis was conducted for each blood SCFA as the dependent variable, the number of individual cows nested in the pasture as a random effect, and time (days 0, 9, 14), group (control, DFA III), its interaction (time*group), and day 0 value of the dependent variable as fixed effects. The estimated mean (least squares [LS] mean) and its 95% confidence interval (CI) were calculated for each time point and the amount of change relative to day 0 for each group was determined. At this time, a comparison test between the DFA III and control groups was performed to determine the amount of change. For each group, a test on day 0 was also performed. A two-sided p < 0.05 was considered statistically significant. All statistical analyses were performed using Statistical Package for the Social Sciences for Windows version 24.0 (IBM Japan, Tokyo, Japan).

## Results

Among the 90 serum samples (Herd 1: 30; Herd 2: 60), four samples from Herd 1 and 15 samples from Herd 2 were excluded from the data analyses because no GC/MS peaks were detected. This may be due to impaired derivatization during sample processing before GC/MS analysis. Finally, data from 26 samples from Herd 1 and 45 samples from Herd 2 were included (analysis included 15 cows in the control group and 14 cows in the DFA III group).

There was no statistically significant difference in the amount of change in acetic, propionic, butyric, 2-methybutanoic, isovaleric, valeric, caproic, and 2-methylhezanelic acids between the control and DAF III groups. However, the values for all these fatty acids in control and DFA III groups showed a significant decrease on day 14 compared with those on day 0.

Table-2 shows the results for control and DFA III supplementation. There were no significant differences in the total change in blood concentrations of SCFAs between groups on day 9 or day 14 (difference in changes [DFA III-control]; day 9: Least squares mean = 2.535 [95% CI: −15.933, 21.003], p = 0.784; day 14: 3.137 [−16.432, 22.707], p = 0.749). Pre-and post-tests in each group showed a significant reduction in fatty acid levels on days 9 and 14 compared to those on day 0 in both the groups (changes from day 0; control: day 9: −21.612 [−39.966, −8.258], p = 0.002; day 14: −47.615 [−61.835, −33.394], p < 0.001; DFA III: day 9: −19.077 [−31.829, −6.325], p = 0.004; day 14: −44.477 [−57.968, −30.986], p < 0.001).

**Table-2:** Concentrations (μM) of short-chain fatty acids in the serum of control and DFA III group animals.

Parameters	Control				DFA III			
	
	LS mean	95%CI		p-value	LS mean	95%CI		p-value
Acetic acid								
Day 0	50.110	44.986	55.235	-	49.241	44.340	54.143	-
Day 9	42.396	37.004	47.789	0.042	44.897	39.775	50.020	0.225
Day 14	30.944	24.936	36.952	<0.001	32.905	27.234	38.577	<0.001
Propionic acid								
Day 0	16.337	14.158	18.516	-	16.702	14.615	18.789	-
Day 9	13.631	11.345	15.917	0.092	14.846	12.667	17.025	0.223
Day 14	9.953	7.394	12.513	<0.001	9.742	7.333	12.150	<0.001
Isobutyric acid								
Day 0	1.108	0.918	1.299	-	0.974	0.794	1.154	-
Day 9	0.863	0.667	1.059	0.072	1.143	0.954	1.333	0.189
Day 14	0.677	0.453	0.902	0.004	0.687	0.478	0.896	0.038
Butyric acid								
Day 0	30.337	27.897	32.778	-	30.858	28.520	33.197	-
Day 9	22.007	19.448	24.566	<0.001	20.348	17.902	22.794	<0.001
Day 14	13.520	10.653	16.388	<0.001	14.971	12.273	17.669	<0.001
2-Methylbutanoic acid								
Day 0	3.047	2.705	3.390	-	2.907	2.581	3.234	-
Day 9	2.481	2.123	2.840	0.025	2.529	2.188	2.869	0.113
Day 14	1.915	1.516	2.313	<0.001	1.789	1.410	2.167	<0.001
Isovaleric acid								
Day 0	1.032	0.902	1.163	-	0.949	0.826	1.073	-
Day 9	0.792	0.656	0.928	0.013	0.804	0.675	0.934	0.109
Day 14	0.576	0.425	0.727	<0.001	0.615	0.471	0.760	<0.001
Valeric acid								
Day 0	4.077	3.751	4.402	-	1.197	1.116	1.277	-
Day 9	3.345	3.003	3.686	0.004	0.829	0.745	0.913	<0.001
Day 14	2.435	2.054	2.816	<0.001	0.636	0.543	0.729	<0.001
Caproic acid								
Day 0	1.185	1.101	1.268	-	1.197	1.116	1.277	-
Day 9	0.880	0.793	0.968	<0.001	0.829	0.745	0.913	<0.001
Day 14	0.671	0.572	0.770	<0.001	0.636	0.543	0.729	<0.001
2-Metylhexanelic acid								
Day 0	1.557	1.386	1.727	-	1.636	1.472	1.800	-
Day 9	1.018	0.840	1.197	<0.001	0.988	0.817	1.158	<0.001
Day 14	0.748	0.547	0.948	<0.001	0.763	0.574	0.951	<0.001
Enhatic acid								
Day 0	0.753	0.696	0.809	-	0.789	0.735	0.844	-
Day 9	0.590	0.531	0.649	<0.001	0.520	0.463	0.576	<0.001
Day 14	0.412	0.344	0.479	<0.001	0.319	0.257	0.381	<0.001

LS mean=Least square mean, 95%CI=95% confidence interval, p*-*value: Versus Day 0, DFA III=Difructose anhydride III

Table-3 shows a substantial difference between DFA III supplementation and control groups. The changes in isobutyric acid concentrations on day 9 were significantly greater in the DFA III group than in the control group (difference in changes [DFA III-control]; LS mean = 0.415 [95% CI: 0.045, 0.785], p = 0.029). Within each group, there was no statistically significant variation between day 0 and day 9 in both groups; however, the values for the control group showed a decreasing tendency (−0.245 [−0.513, 0.023], p = 0.072), whereas those for the DFA III group showed an increasing tendency (0.170 [−0.086, 0.425], p = 0.189).

**Table-3:** Data for change in the concentrations of short-chain fatty acids between control and DFA III group animals.

Parameters	Difference (DFA III-control)			

	LS mean	95%CI		p-value
Acetic acid				
⊿Day 9 (from 0)	3.370	−6.898	13.638	0.513
⊿Day 14 (from 0)	2.830	−8.047	13.708	0.604
Propionic acid				
⊿Day 9 (from 0)	0.850	−3.519	5.219	0.698
⊿Day 14 (from 0)	−0.577	−5.205	4.052	0.804
Isobutyric acid				
⊿Day 9 (from 0)	0.415	0.045	0.785	0.029*
⊿Day 14 (from 0)	0.145	−0.248	0.538	0.464
Butyric acid				
⊿Day 9 (from 0)	−2.180	−7.069	2.709	0.375
⊿Day 14 (from 0)	0.929	−4.254	6.112	0.721
2-Methylbutanoic acid				
⊿Day 9 (from 0)	0.188	−0.494	0.869	0.583
⊿Day 14 (from 0)	0.014	−0.709	0.737	0.969
Isovaleric acid				
⊿Day 9 (from 0)	0.095	−0.163	0.353	0.463
⊿Day 14 (from 0)	0.123	−0.151	0.396	0.373
Valeric acid				
⊿Day 9 (from 0)	−0.064	−0.231	0.104	0.451
⊿Day 14 (from 0)	−0.047	−0.225	0.131	0.600
Caproic acid				
⊿Day 9 (from 0)	−0.064	−0.231	0.104	0.451
⊿Day 14 (from 0)	−0.047	−0.225	0.131	0.600
2-Metylhexanelic acid				
⊿Day 9 (from 0)	−0.110	−0.452	0.231	0.520
⊿Day 14 (from 0)	−0.065	−0.427	0.297	0.721
Enhatic acid				
⊿Day 9 (from 0)	−0.107	−0.217	0.003	0.056
⊿Day 14 (from 0)	−0.129	−0.246	−0.013	0.031*

LS mean=Least square mean, 95%CI=95% confidence interval, p*-*value: DFA III vs. Control,

* Significant differences between DFA III and control groups

The decrease in enanthic acid concentration on day 14 was significantly greater in the DFA III group than in the control group (difference in changes [DFA III-control]; LS mean = −0.129 [95%CI: −0.246, −0.013], p = 0.031). Within each group, a statistically significant decrease was observed on day 14 compared with that on day 0 (changes from day 0; control: −0.341 [−0.426, −0.256], p < 0.001; DFA III: −0.470 [−0.550, −0.390], p < 0.001).

## Discussion

Previously, with experimental animals and humans, oligosaccharides, such as DFA III and DFA IV, extracted from inulin were reported to increase the number of health-promoting bacteria and decrease harmful bacteria in the host gastrointestinal tract and increase the concentrations of SCFAs, including acetic acid, which may alter the intestinal microbiota toward a healthier composition [8, 9]. In addition, treatment of such oligosaccharides was proposed to restore the impaired epithelial barrier functions in the small intestinal epithelial cells [8, 28]. We have also reported the effects of DFA III supplementation on calf/cattle, where we showed that supplementing DFA III with milk replacer reduced the onset of diseases, such as fever and diarrhea, during the pre-weaning period [17, 18], and the effect of supplementing DFA III on feed-related epithelial barrier functions to reduce the absorption of mycotoxins, such as ZEN and STC, from the intestinal tract by measuring their urinary concentrations [19, 20]. As a stepping-stone to elucidate the effects of DFA III supplementation on cattle health and intestinal defense function, in this study, we aimed to confirm whether the addition of DFA (40 g/days) to the diet increased the production of fatty acids in the intestinal tract and caused a difference in the serum concentrations of SCFAs after absorption from the intestinal tract. The results of the present study indicate that although there was no significant difference in the total SCFA concentrations between the DFA III and control groups, DFA III supplementation may affect the composition of SCFAs produced in the intestinal tract. We observed significant reductions in serum SCFA levels on days 9 and 14 compared with those on day 0 in both groups. The heifers used in this study were newly introduced to each herd for fattening immediately before the start of the experiment. Therefore, it is conceivable that the difference in the composition of the feed before and after the introduction led to a decrease in the concentrations of SCFAs at the beginning of DFA III supplementation and during the subsequent test period.

Difructose anhydride III is an indigestible oligosaccharide enzymatically synthesized from inulin [21]. *In vitro* experiments conducted on the small intestines of rats [29, 30] and on the duodena of cows [26] have shown that the absorption of calcium through the paracellular pathway can be accelerated by agents that act on tight junctions. Difructose anhydride III promotes paracellular transport by reducing transepithelial electrical resistance and by enhancing the transport of paracellular markers [31, 32], with alterations in claudin-1, a component of tight junctions and actin filaments in Caco-2 cells [32]. Tight junctions are crucial in paracellular nutrient transport and barrier functions in the intestine. Previously, we reported that DFA III, when supplemented in the diet of cattle, could be successfully used as a mycotoxin-mitigating substance, against mycotoxins, such as zearalenone [19] and sterigmatocystin [20], due to the known beneficial effects of prebiotic DFA III on the integrity of the intestinal barrier and gut health. Difructose anhydride III has been suggested to exhibit extremely low degradability and fermentability by microflora in the rumen, which strongly suggests that it bypasses the rumen and reaches the intestinal tract [33]. Recent findings in broilers suggested that crosstalk occurs between nutrients and epithelial barrier function through the dynamic regulation of tight junctions and enhanced intestinal barrier function mediated by DFA IV [8]. Therefore, in this study, we examined whether dietary DFA III changes the serum concentrations of SCFAs, reflecting the fermentation of SCFAs in the intestinal tract and their absorption from the intestine. The results indicated that although there were no significant differences in change in total SCFAs between groups on day 9 or day 14, the changes in the concentration of isobutyric acid on day 9 were significantly greater in the DFA III group than in the control group (p = 0.029). In addition, within each group, there was no statistically significant variation between days 0 and 9 in both groups; however, the control group showed a tendency of decrease in concentration (p = 0.072) and the DFA III group showed a tendency of increase in concentration (p = 0.189). In addition, the decrease in enanthic acid on day 14 was significantly greater in the DFA III group than in the control group (p = 0.031). SCFAs, primarily acetate, butyrate, and propionate, are predominantly produced by fermenting dietary fibers in the gut using anaerobic bacteria and have been shown to possess anti-inflammatory potential by inhibiting the production of inflammatory cytokines. They help reduce LPS-induced pathological damage to the intestine [8] or mammary gland [28]. Based on a recent report, the serum concentration of isobutyric acid in dairy cattle (n = 18) was in the 1.59–18.63 mM/L range; the large inter-cow variation in SCFA content suggests that SCFA molecules may be closely associated with the metabolism in individual animals, as all animals were managed in the same herd with the same feeding regime [24]. In this study, lower concentrations of isobutyric acid than those reported previously were observed, possibly due to differences in dietary feed quality and quantity between dairy Holstein cows and JB heifers. As mentioned above, DFA III has been suggested to have extremely low degradability and fermentability by microflora in the rumen, which strongly suggests that it bypasses the rumen and reaches the intestinal tract [33]. Li *et al*. [24] also reported that the isobutyric acid concentration in ruminal fluids after dietary inulin (original source of DFA III) supplementation did not change. Iso-acids, such as isobutyric acid, are formed during the fermentation of amino acids, and higher ruminal concentrations of iso-acids have been reported after dietary supplementation with yeast, which may stimulate the growth of amylolytic bacteria that preferentially use peptides and amino acids due to their proteolytic activity [34]. Alternatively, from the results of monensin supplementation in dairy cattle, a significant decrease in enenthic acid concentrations in rumen fluids was reported, suggesting a greater development of cellulolytic bacteria in the rumen [35]. Therefore, it may be speculated that our results reflect the changes in bacterial flora in the intestine after the fermentation of DFA III. Further research is necessary to elucidate the effects of DFA III on cattle.

## Conclusion

It is concluded that DFA III supplementation for at least 2 weeks affects the trends of blood SCFA concentrations in cattle. As we previously reported, oligosaccharides, such as DFA III, that are now widely used as prebiotics, can be successfully used as mycotoxin-mitigating substances with beneficial effects on intestinal barrier integrity and gut health [20]. Our results support the effects of DFA III on the intestinal environment and barrier function. By further accumulating and integrating field- and *in vitro* data, new mycotoxin control methods that can be applied to livestock production sites will be developed.

## Authors’ Contributions

TWP, SU, OY, and MiT: Conceived and designed the study. SU, EK, OY, MO, DP, and MiT: Collected the samples and performed formal analysis. TWP, SU, EK, KT, HH, DM, OY, OSW, MO, MT, and MIT: Conducted the literature review and prepared tables. TWP, MT, and MiT: Drafted the manuscript, reviewed, and edited. All authors have read, reviewed, and approved the final manuscript.

## References

[1] Heinrichs A.J, Jones C.M, Heinrichs B.S (2003). Effects of mannan oligosaccharide or antibiotics in neonatal diets on health and growth of dairy calves. J. Dairy Sci.

[2] Timmerman H.M, Mulder L, Everts H, van Espen D.C, van der Wal E, Klaassen G, Rouwers S.M, Hartemink R, Rombouts F.M, Beynen A.C (2005). Health and growth of veal calves fed milk replacers with or without probiotics. J. Dairy Sci.

[3] Jouany J.P, Morgavi D.P (2007). Use of “natural”products as alternatives to antibiotic feed additives in ruminant production. Animal.

[4] Franklin S.T, Newman M.C, Newman K.E, Meek K.I (2005). Immune parameters of dry cows fed mannan oligosaccharide and subsequent transfer of immunity to calves. J. Dairy Sci.

[5] Donovan D.C, Franklin S.T, Chase C.C.L, Hippen A.R (2002). Growth and health of Holstein calves fed milk replacers supplemented with antibiotics or Enteroguard. J. Dairy Sci.

[6] Fleige S, Preißinger W, Meyer H.H.D, Pfaffl M.W (2007). Effect of lactulose on growth performance and intestinal morphology of pre-ruminant calves using a milk replacer containing *Enterococcus faecium*. Animal.

[7] Heinrichs A.J, Jones C.M, Elizondo-Salazar J.A, Terrill S.J (2009). Effects of a prebiotic supplement on health of neonatal dairy calves. Livest. Sci.

[8] Lee S.I, Kim I.H (2018). Difructose dianhydride improves intestinal calcium absorption, wound healing, and barrier function. Sci. Rep.

[9] Wang Y, Nan X, Zhao Y, Jiang L, Wang H, Zhang F, Hua D, Liu J, Yao J, Yang L, Luo Q, Xiong B (2021). Dietary supplementation of inulin ameliorates subclinical mastitis via regulation of rumen microbial community and metabolites in dairy cows. Microbiol. Spectr.

[10] Fink-Gremmels J (2008). The role of mycotoxins in the health and performance of dairy cows. Vet. J.

[11] Liu G, Yan T, Wang J, Huang Z, Chen X, Jia G, Wu C, Zhao H, Xue B, Xiao L, Tang J (2013). Biological system responses to zearalenone mycotoxin exposure by integrated metabolomic studies. J. Agric. Food Chem.

[12] Akbari P, Braber S, Alizadeh A, Verheijden K.A, Schoterman M.H, Kranevald A.D, Garssen J, Fink-Gremmels J (2015). Galacto-oligosaccharides protect the intestinal barrier by maintaining the tight junction network and modulating the inflammatory responses after a challenge with the mycotoxin deoxynivalenol in human Caco-2 cell monolayers and B6C3F1 mice. J. Nutr.

[13] Akbari P, Braber S, Varasteh S, Alizadeh A, Garssen J, Fin-Gremmels J (2017). The intestinal barrier as an emerging target in the toxicological assessment of mycotoxins. Arch. Toxicol.

[14] Akbari P, Fink-Gremmels J, Willems R.H.A.M, Difilippo E, Schols H.A, Schoterman M.H.C, Garssen J, Braber S (2017). Characterizing microbiota-independent effects of oligosaccharides on intestinal epithelial cells:Insight into the role of structure and size:Structure-activity relationships of non-digestible oligosaccharides. Eur. J. Nutr.

[15] Yokota A, Hirayama S, Enomoto K, Miura Y, Tomita F (1991). Production of inulin fructotransferase (depolymerizing) by *Arthrobacter* spp. H65-7 and preparation of DFA III from inulin by the enzyme. J. Fermen. Bioengin.

[16] Tamura A, Shiomi T, Tamaki N, Shigetmatsu N, Tomita F, Hara H (2004). Comparative effect of repeated ingestion of difructose anhydride III and palatinose on the induction of gastrointestinal symptoms in humans. Biosci. Biotechnol. Biochem.

[17] Matsumoto D, Takagi M, Hasunuma H, Fushimi Y, Ohtani M, Sato T, Okamoto K, Shahada F, Tanaka T, Deguchi E (2009). Effects of oral administration of difructose anhydride III on selected health and blood parameters of group-housed Japanese black calves during the pre-weaning period. Asian. Aust. J. Anim. Sci.

[18] Takagi M, Hasunuma H, Matsumoto D, Obi T, Takase K, Ohtani M, Sato T, Watanabe U, Okamoto K (2011). Effects of daily oral administration of difructose anhydride III on health status, blood parameters and faecal shedding of coliform bacteria of Japanese black calves during the pre-weaning period. Anim. Nutr. Feed Technol.

[19] Toda K, Uno S, Kokushi E, Shiiba A, Hasunuma xH, Matsumoto D, Ohtani M, Yamato O, Shinya U, Wijayagunawardane M, Fink-Gremmels J, Taniguchi M, Takagi M (2018). Fructo-oligosaccharide. (DFA III) feed supplementation for mitigation of mycotoxin exposure in cattle-clinical evaluation by a urinary zearalenone monitoring system. Toxins (Basel).

[20] Sasazaki N, Uno S, Kokushi E, Toda K, Hasunuma H, Matsumoto D, Miyashita A, Yamato O, Okawa H, Ohtani M, Fink-Gremmels J, Taniguchi M, Takagi M (2021). Mitigation of sterigmatocystin exposure in cattle by difructose anhydride III feed supplementation and detection of urinary sterigmatocystin and serum amyloid A concentrations. Arch. Anim. Breed.

[21] Kikuchi H, Nagura T, Inoue M, Kishida T, Sakurai H, Yokota A, Asano K, Tomita F, Sayama K, Senba Y (2004). Physical, chemical and physiological properties of difructose anhydride III produced from inulin by enzymatic reaction. J. Appl. Glycosci.

[22] Tamura A, Nino H, Minobe T, Raneva V.G, Shigematsu N, Hara H, Kishida T, Ebihara K (2006). Difructose anhydride III does not contribute to body energy accumulation in rats. Biosci. Biotechnol. Biochem.

[23] Chibisa G.E, Beauchemin K.A, Koenig K.M, Penner G.B (2020). Optimum roughage proportion in barley-based feedlot cattle diets:Total tract nutrient digestibility, rumination, ruminal acidosis, short-chain fatty absorption, and gastrointestinal tract barrier function. J. Anim. Sci.

[24] Li C, Liu Z, Bath C, Marett L, Pryce J, Rochfort S (2022). Optimised method for short-chain fatty acid profiling of bovine milk and serum. Molecules.

[25] Sato T, Nakai T, Sadoya H, Ohtani M, Hanada M, Okamoto M (2007). Effect of difructose anhydride III on hypocalcemia in dairy cows after calving. Anim. Sci. J.

[26] Teramura M, Wynn S, Reshalaitihan M, Kyuno W, Sato T, Ohtani M, Kawashima C, Hanada M (2015). Supplementation with difructose anhydride III promotes passive calcium absorption in the small intestine immediately after calving in dairy cows. J. Dairy Sci.

[27] Furuhashi T, Sugitate K, Nakai T, Jikumaru Y, Ishihara G (2018). Rapid profiling method for mammalian faeces short-chain fatty acids by GC-MS. Anal. Biochem.

[28] Ali I, Raza A, Ahmad M.A, Li L (2022). Nutrient sensing mechanism of short-chain fatty acids in mastitis control. Microb. Pathog.

[29] Suzuki T, Hara H, Kasai T, Tomita F (1998). Effects of difructose anhydride III on calcium absorption in small and large intestines of rats. Biosci. Biotechnol. Biochem.

[30] Mineo H, Hara H, Shigematsu N, Okuhara Y, Tomita F (2002). Melibiose, difructose anhydride III and difructose anhydride IV enhance net calcium absorption in rat small and large intestinal epithelium by increasing the passage of tight junctions *in vitro*. J. Nutr.

[31] Suzuki T, Hara H (2004). Various non-digestible saccharides open a paracellular calcium transport pathway with the induction of intracellular calcium signalling in human intestinal Caco-2 cells. J. Nutr.

[32] Suzuki T, Hara H (2006). Difructose anhydride III and sodium caprate activate paracellular transport via different intracellular events in Caco-2 cells. Life Sci.

[33] Sato T, Kikuchi H, Nakai T, Sadoya H, Hanada M, Okamoto M (2006). Effect of ruminal bacteria on degradability of difructose anhydride III. Anim. Sci. J.

[34] Diaz T.G, Branco A.F, Jacovaci F.A, Jobim C.C, Daniel J.L.P, Bueno A.V.I, Ribeiro M.G (2018). Use of live yeast and mannanoligosaccharides in grain-based diets for cattle:Ruminal parameters, nutrient digestibility, and inflammatory response. PLoS One.

[35] Mammi L.M.E, Guadagnini M, Mechor G, Cainzos J.M, Fusaro I (2021). The use of monensin for ketosis prevention in dairy cows during the transition period:A systematic review. Animals (Basel).

